# Interpretation of reproductive hormones before, during and after the pubertal transition—Identifying health and disordered puberty

**DOI:** 10.1111/cen.14578

**Published:** 2021-08-08

**Authors:** Sasha R. Howard

**Affiliations:** ^1^ Centre for Endocrinology, William Harvey Research Institute, Barts and the London School of Medicine and Dentistry Queen Mary University of London London UK

**Keywords:** delayed puberty, gonadotropins, gonadotropin‐releasing hormone, precocious puberty, puberty

## Abstract

Puberty is a process of transition from childhood to adult reproductive capacity, governed by the reactivation of the hypothalamic–pituitary–gonadal axis after a long period of dormancy in mid‐childhood. As such, the reproductive hormones are in a state of flux during the adolescent years, and interpretation of both the onset of healthy, concordant puberty and the differentiation of precocious, delayed or disordered puberty, can be challenging. This review is focused on the description of the endocrine axes in healthy puberty and the markers of disorders of puberty that can aid diagnosis and management for patients with these conditions. It will cover the hypothalamic, pituitary and gonadal hormone systems, the dynamic changes that occur during puberty, conditions leading to precocious, delayed or absent puberty and other syndromes with disordered puberty, and the biochemical diagnosis of these different disorders of puberty.

## 1 INTRODUCTION

**Figure 1:**
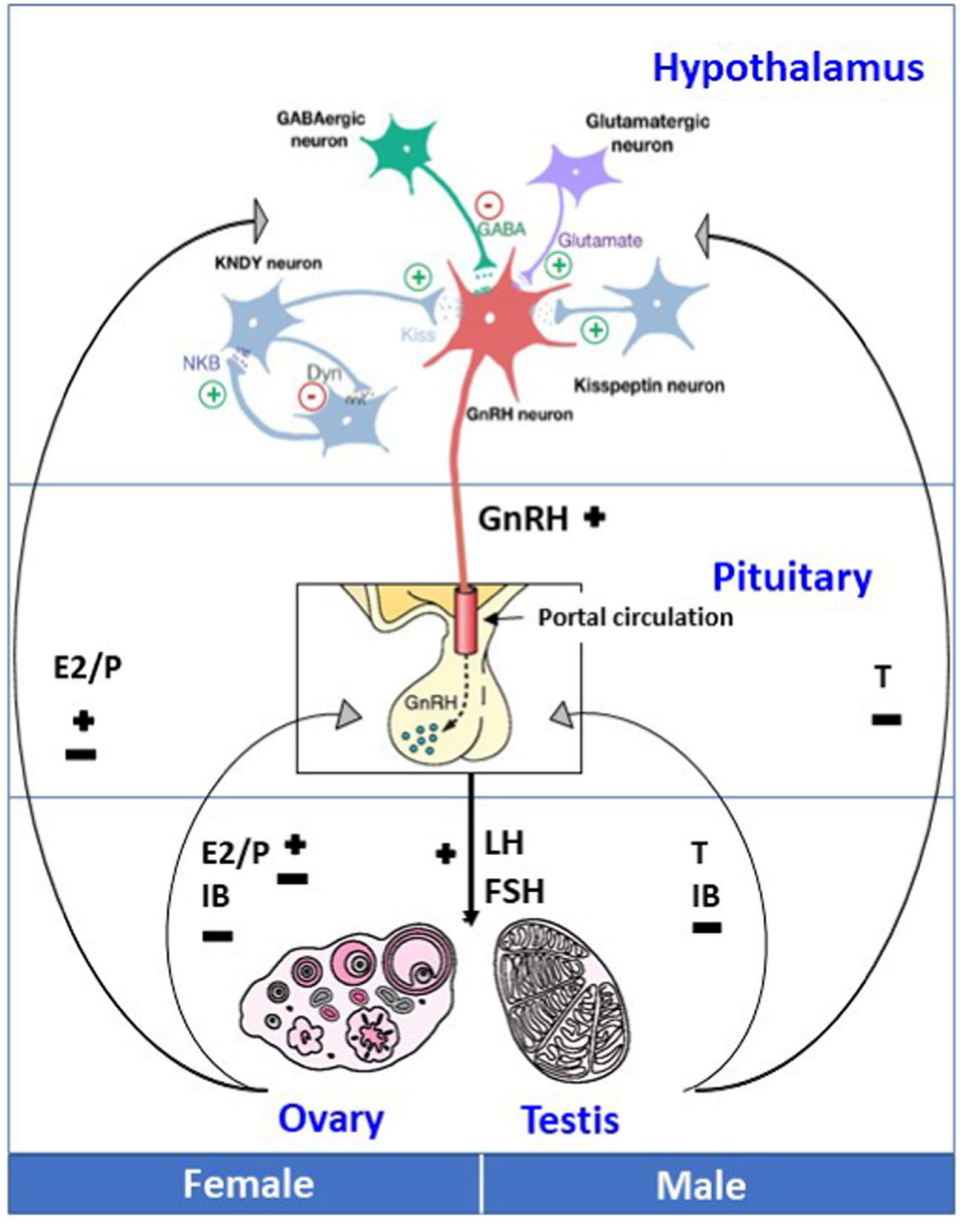
Schematic of the hypothalamic–pituitary–gonadal axis. GnRH production from hypothalamic GnRH neurons is regulated by kisspeptin, neurokinin, dynorphin (KNDy) and other upstream signalling, including GABAergic and glutamatergic neurons. GnRH travels via the portal circulation to the anterior pituitary where it stimulates LH and FSH production. These gonadotropins act in males on the testes and in females on the ovaries to produce the sex steroids testosterone and estradiol/progesterone (E2/P), respectively. There is negative feedback from inhibin B (IB) on the pituitary, and a mix of negative and positive feedback from sex steroids on the pituitary and on the hypothalamus via kisspeptin neurons. −, negative feedback; +, positive feedback; FSH, follicle‐stimulating hormone; GnRH, gonadotropin‐releasing hormone; LH, luteinizing hormone [Color figure can be viewed at wileyonlinelibrary.com]

**Figure 2:**
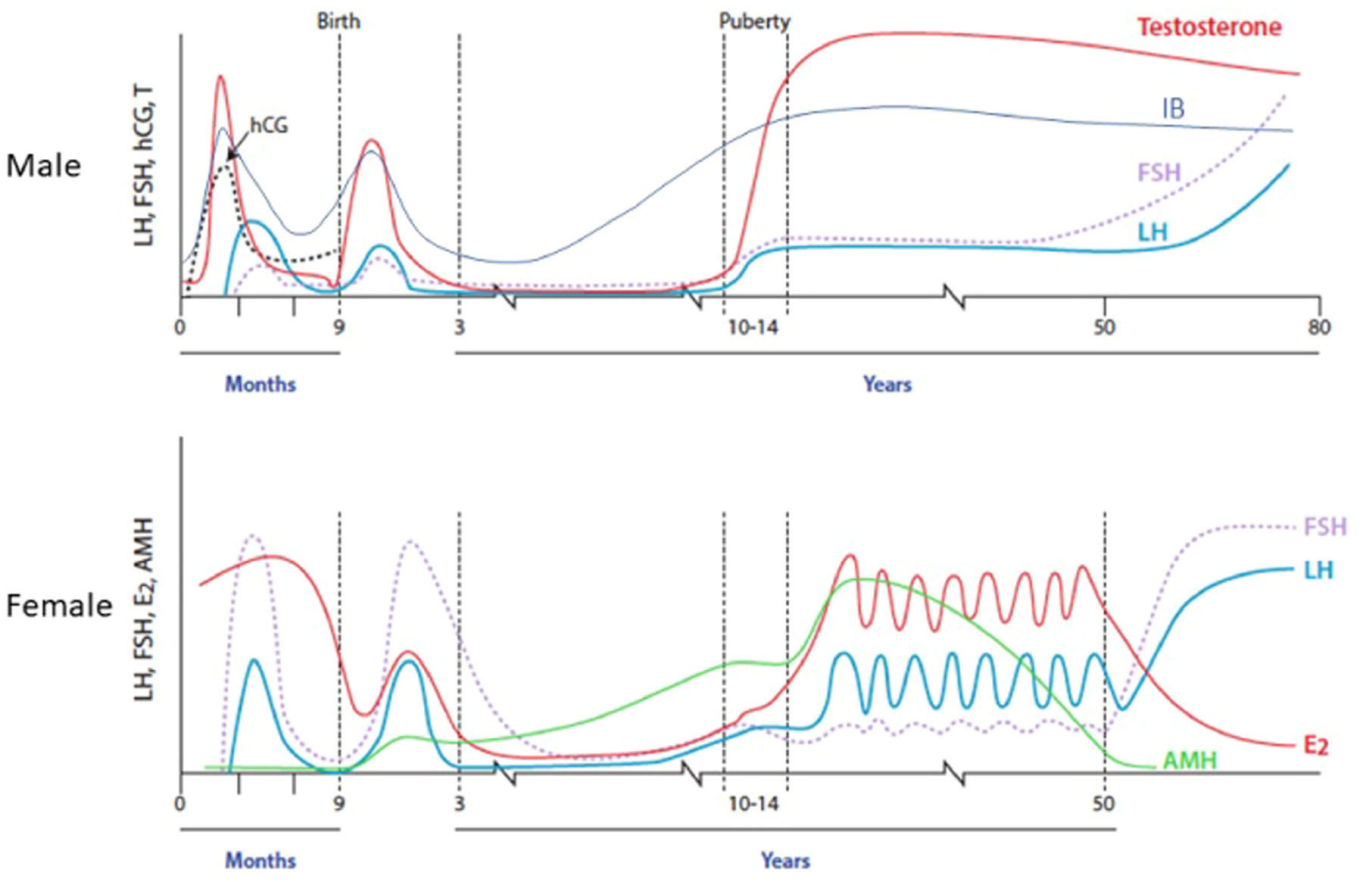
The HPG axis during foetal and postnatal life. Circulating concentrations of gonadotropins (hCG, FSH, LH) and testosterone and inhibin B (IB) during the prenatal and postnatal period in males (top panel), and gonadotropins, estradiol and anti‐Müllerian hormone in females (lower panel). FSH, follicle‐stimulating hormone; hCG, human chorionic gonadotropin; HPG, hypothalamo–pituitary–gonadal; LH, luteinizing hormone. Modified from Huhtaniemim et al.^2^ and Kelsey et al.^3^[Color figure can be viewed at wileyonlinelibrary.com]

Puberty is the transitional period in adolescence that leads to the attainment of reproductive capacity. The physical, psychological and emotional changes that occur are the consequence of sex hormone synthesis by the gonads, under the control of the hypothalamic–pituitary axis (Figure 1). The activation of pulsatile gonadotropin‐releasing hormone (GnRH) secretion from the hypothalamus is the endocrine hallmark of the onset of puberty. The development of this hypothalamic–pituitary axis starts in foetal life, with rising gonadotropin concentrations from the end of the first trimester, although these are lower in males than in females and decline by birth in both sexes.^1^ The axis is reactivated in early infant life, in a period named ‘mini‐puberty’, peaking between 1 week and 3 months of age in both sexes but with a later peak and longer tail in female infants. There is then a prolonged period of dormancy in young children between the approximate ages of 2 and 8–9 years (Figure 2).

Puberty is initiated by the third activation of the hypothalamo–pituitary–gonadal (HPG) axis, after this childhood quiescence. The hypothalamic neurons that secrete GnRH are at the heart of a complex neuroendocrine network composed of kisspeptin, neurokinin B and dynorphin‐expressing neurons, as well as glial cells, such as tanycytes, astrocytes and ependymal cells.^4^ These neurons and glial cells act together to stimulate the pulsatile secretion of GnRH. This activation results in increased luteinizing hormone (LH) and follicle‐stimulating hormone (FSH) release from the anterior pituitary, which act on the gonads to stimulate their development, gametogenesis and sex steroid and gonadal peptide hormone production.

## 2 REPRODUCTIVE HORMONES OF PUBERTY

### 2.1 Hypothalamic–pituitary hormones

#### 2.1.1 Gonadotropin‐releasing hormone

GnRH is the master hormone of puberty, via which activation of the HPG axis is controlled.^5^ GnRH is a decapeptide (pGlu‐His‐Trp‐Ser‐Tyr‐Gly‐Leu‐Arg‐Pro‐Gly.NH_2_), which is enzymatically processed from a large 69‐amino acid prohormone precursor. GnRH is synthesized in specialized neurons of the hypothalamus, but is not localized to any one discrete nucleus and is also found outside of the hypothalamus in the hippocampus, cingulate cortex and olfactory bulbs.

GnRH release is coordinated through a balance of inhibitory (e.g., GABA) and excitatory (e.g., kisspeptin, glutamate) neuronal and glial inputs (Figure 1).^6^ GnRH is secreted in synchronized pulses into the hypothalamic–pituitary portal system from the nerve endings of about 1000 neurons.^7^ The synchrony of these GnRH pulses across multiple GnRH neurons is a complex process, as there is spontaneous electrical activity of the neurons, as well as autocrine regulation through the GnRH receptor. It is likely that the upstream regulation by key peptides, kisspeptin, neurokinin B and dynorphin, produced by neurons in the arcuate nucleus, is ultimately responsible for coordinating GnRH pulse generation.^8^ These three peptides act on GnRH neurons to modulate their activity via cell surface receptors and their coordinated action is known as the ‘KNDy’ model of pulse generation. These pulses occur every 30–120 min and stimulate the processing and secretion of gonadotropins from pituitary gonadotrope cells.^9^

#### 2.1.2 Gonadotropins

LH and FSH are produced by the gonadotrope cells of the anterior pituitary in response to GnRH stimulation.^10^ GnRH travels to the anterior pituitary via the hypothalamo–hypophyseal portal circulation (Figure 1) and there binds to its receptor (GnRHR), a typical G‐protein‐coupled receptor, on the gonadotrope cell surface. Activation of GnRHR triggers an increase in intracellular calcium concentration and downstream phosphorylation of protein kinase C. Gonadotropes are found throughout the anterior pituitary gland and abut upon the capillary basement membranes to facilitate access to the bloodstream. The same gonadotrope cells produce both LH and FSH, and these cells are small in diameter when the HPG axis is dormant but increase in size in response to GnRH stimulation.

Each GnRH pulse stimulates a pulse of LH, but FSH pulses are less clearly correlated with GnRH release, due to the longer circulatory half‐life of FSH. LH is secreted rapidly in response to a bolus of GnRH from pre‐existing pools of LH, leading to a rise in circulating LH within minutes, as well as more slowly after further LH processing. There is also a differential sensitivity between LH and FSH response to GnRH,^11^ such that a partial decrease in the activity of the GnRHR can lead to reduced LH and sex steroid hormone production with relatively preserved FSH concentrations.^12^ While stimulation by GnRH in an intermittent or pulsatile manner leads to increased gonadotropin secretion, a continuous infusion of GnRH results in a decrease in LH and FSH secretion and a downregulation of the GnRH receptors on the pituitary gonadotrope cells.^13^ These alterations in the GnRH receptor have an important role in regulating gonadotrope function.

FSH and LH are glycoproteins that share an identical α‐subunit but have distinct 115 amino acid long β‐subunits that confer specificity. There are a small number of published cases of mutations in the gonadotropin‐specific β‐subunits, including an inactivating mutation of beta LH causing pubertal failure with the absence of Leydig cells of the testes,^14^ in contrast to inactivating mutations of beta FSH, which lead to lack of follicular maturation and amenorrhoea in females and failure of spermatogenesis in males, although there is some phenotypic variability.^15^ Pubertal maturation in women, including breast development and menarche (indicating sufficient oestrogen production for a mitogenic action on the breast tissue and endometrium), can occur in a state of LH deficiency, although LH secretion is obligatory for ovulation. These observations imply that LH is essential for the maturation of Leydig cells and steroidogenesis in men and that its primary role in women is to induce ovulation^16^ (Table 1).

**Table 1:** Clinical features of isolated gonadotropin deficiency in males and females

Isolated LH deficiency^17^		Isolated FSH deficiency^18^	
General features	Fertility	General features	Fertility
Males			
Under‐virilisation or delayed puberty, eunuchoid body proportions, gynaecomastia	Comparatively large testes volumes for serum testosterone, +/− oligospermia with arrested spermatogenesis	Normal or delayed puberty, small, soft testes	Azoospermia, or oligospermia with abnormal morphology

**Isolated LH deficiency** ^19^		**Isolated FSH deficiency** ^20^	
General features	Fertility	General features	Fertility
Females			
Normal pubertal development with menarche	Oligomenorrhoea with lack of ovulation	Delayed or arrested breast and uterine development	Amenorrhoea, with ovarian follicular development arrested at preantral stage

Abbreviations: FSH, follicle‐stimulating hormone; LH, luteinizing hormone.

### 2.2 Gonadal hormone production

#### 2.2.1 Sex steroids

##### 2.2.1.1 Males

The Leydig cells of the testes synthesize testosterone from cholesterol through a series of enzymatic conversions stimulated by the binding of LH its receptor, the luteinizing hormone/chorionic gonadotropin receptor (LHCGR) on the Leydig cell membrane. LHCGR numbers and downstream responsiveness decrease after exposure to LH for at least 24 h, leading to insensitivity to LH after daily injections of LH or human chorionic gonadotropin (hCG) compared to alternate day injections. Thus, hCG or LH must be administered at 2–3 day intervals to eliminate such downregulation when assessing the response of the testes to LH‐like stimulation.

The majority of circulating testosterone is bound to sex‐hormone‐binding globulin (SHBG) or albumin, with the remaining ‘free’ testosterone considered to be responsible for biological activity.^2^ Testosterone is modulated by prereceptor metabolism through conversion, either by 5α‐reductase type 2 (a surface enzyme located on the genital skin and elsewhere) to dihydrotestosterone or by aromatase (CYP19A1) to estradiol. Testosterone and dihydrotestosterone both bind to the androgen receptor but with a greater affinity for dihydrotestosterone.^21^ The testosterone/dihydrotestosterone‐receptor complex attaches to the steroid‐responsive region of genomic DNA to initiate androgen‐dependent transcription and translation.

Dihydrotestosterone is responsible for virilisation of the external genitalia and for most of the secondary sexual characteristics of puberty, including phallic growth, prostate enlargement, androgen‐induced hair loss and beard growth. Testosterone promotes muscle development and stimulates enzymatic activity in the liver and haemoglobin synthesis. Testosterone sends negative feedback to regulate LH secretion, directly or after conversion to estradiol, through suppression of GnRH secretion via kisspeptin neurons at the hypothalamic level and by acting directly on gonadotropin synthesis in the pituitary gland (Figure 1).^22^ Estradiol aromatized from testosterone stimulates bone maturation at the epiphyseal plate.

FSH binds to specific receptors on the cell surface of Sertoli cells resulting in a similar intracellular cascade to that produced by LH binding on Leydig cells. FSH causes an increase in the mass of seminiferous tubules and supports the development of sperm, particularly in the early stages of meiosis.^23^ Complete spermatogenesis and spermiogenesis is dependent on both FSH and intratesticular concentrations of testosterone and DHT.^24^

##### 2.2.1.2 Females

In females, LH drives the conversion of cholesterol to androstenedione in theca cells of the ovary. Androstenedione is then converted to estradiol by CYP19A1 in granulosa cells, under the stimulation of FSH, in what is known as the ‘2 cell‐2 gonadotropin’ model of follicular steroidogenesis. Estradiol is the main active oestrogen in humans and circulates bound to SHBG. Estradiol promotes the development of breast and uterine tissues and influences the distribution of adipose tissue and bone mineral accretion. Low concentrations of estradiol are difficult to measure in standard assays.

### 2.3 Sex steroid feedback on the hypothalamus and pituitary

Once the HPG axis is activated by GnRH stimulation, and sex steroids and gonadal peptide concentrations rise, these then provide negative feedback inhibition to the hypothalamus and pituitary to decrease pituitary LH and FSH secretion. This is seen most profoundly in individuals with gonadal dysgenesis who have high concentrations of LH and FSH during infancy and adolescence.^25^, ^26^

In healthy females, positive feedback by estradiol also occurs from mid‐puberty onwards, a process that is necessary for ovulation. This requires an adequate pool of LH for release from the pituitary and priming of the ovarian follicle to reach a sufficient size to produce adequate oestrogen, both of which are kisspeptin‐dependent processes.^27^ Estradiol also increases pituitary gland sensitivity to GnRH, which, in addition to its action to increase GnRH pulse frequency via positive feedback on the hypothalamic kisspeptin neurons, increases LH secretion. The increase in estradiol also suppresses FSH within the ovary to allow luteinization of the dominant follicle in the presence of LH with subsequent ovulation. Progesterone, the dominant female hormone during the luteal phase, in contrast, slows LH pulse frequency,^28^ inhibits proliferation and stimulates differentiation of endometrial cells.

#### 2.3.1 Gonadal peptide hormones

##### 2.3.1.1 Inhibin and activin

Inhibin B is a heterodimeric glycoprotein member of the transforming growth factor‐β family. In males, it is produced exclusively by the Sertoli cells of the testes, and in females by the ovarian granulosa cells and the placenta. Serum inhibin‐B concentrations vary during childhood in response to gonadotropin secretion.^29^ In boys during the mini‐puberty, when Sertoli cells proliferate but do not mature, serum inhibin‐B concentrations increase to similar or higher concentrations to those observed in adolescent boys. These levels are sustained until the age of 18–24 months, after which they decline to lower but readily measurable concentrations (Figure 2).^30^ Inhibin B concentrations rise again early in puberty, reaching peak concentrations at Tanner stage G2, but then plateau.^31^ In girls, from birth to 6 months, inhibin B levels are approximately 50% lower than at female puberty, when they peak at approximately 50% of the levels seen in male pubertal subjects.^32^ Between mini‐puberty and puberty inhibin B levels in girls are low. Inhibin B exerts negative feedback on the secretion of FSH from the pituitary gland, which is thought to also contribute to the observed difference in response of LH and FSH to GnRH stimulation. Thus, the absence of inhibin due to gonadal failure causes a greater rise in serum FSH than LH in pubertal and adult subjects. Activin, a subunit of inhibin, has an opposite effect to inhibin B, acting to stimulate the secretion of FSH from the pituitary gland.

##### 2.3.1.2 Anti‐Mullerian hormone

Anti‐Müllerian hormone (AMH) belongs to the same family of transforming growth factor‐β as inhibin B. In males, it is produced from the testicular Sertoli cells from the time of testicular differentiation to puberty, and in females, it is secreted by the ovarian granulosa cells from birth until menopause.^33^, ^34^ In healthy males, AMH is high in the foetus and newborn, peaking at mini‐puberty around 2 months of age and then decreases by the age of 1 year.^30^ Patients with dysgenetic gonads have low serum AMH while values are elevated in tumours of the Sertoli or granulosa cells. Undetectable AMH and inhibin B are characteristic of congenital anorchia but may also be seen in males with severe hypogonadotropic hypogonadism. A similar pattern in AMH concentrations during the first months of life has also been reported in infant girls, but the concentrations in girls are significantly lower.^30^ AMH plateaus during puberty as a sign of androgen action. In girls, concentrations are a marker of ovarian granulosa cell function and are considered a novel marker for follicular reserve. This is because AMH is produced mainly by growing follicles, 5–8‐mm diameter, while larger follicles selected for dominance have a marked reduction in their AMH secretion.^35^ This has importance, for example, in Turner syndrome, for assessment of potential reproductive capacity.^36^

##### 2.3.1.3 Insulin‐like 3

Insulin‐like 3 (INSL3) is produced by the Leydig cells and is important in the male foetus for testicular descent. Concentrations increase in males at puberty and correlate with LH and testosterone. In subjects with Klinefelter syndrome, lower INSL3 concentrations indicate Leydig cell dysfunction from mid‐puberty onward.^37^ In females, INSL3 is produced by ovarian theca cells of growing antral follicles^38^ and is important for androstenedione synthesis, and therefore oestrogen production. It is not detectable in girls until puberty.^39^ Knockout studies in mice lead to partial infertility and concentrations are increased in polycystic ovarian syndrome (PCOS) and decreased in women with premature ovarian insufficiency.^40^

### 2.4 Dynamic hormonal changes during pubertal maturation

After periods of activity of the HPG axis in utero, and then during mini‐puberty, the axis becomes dormant between the ages of approximately 2 and 8–9 years (Figure 2). The suppression of the axis is not absolute as LH pulsatility is detectable during this stage using ultrasensitive assays, but pulses are of low amplitude and infrequent. Likewise, testosterone and estradiol are measurable in the circulation using sensitive assays, thus demonstrating low but definite activity of the prepubertal gonads. Notably, serum gonadotropin concentrations are low in early‐mid childhood in the majority of children with gonadal disorders, demonstrating that it is central inhibition rather than negative feedback from sex steroid production that suppresses gonadotropin secretion during this period.

The increasing amplitude of GnRH pulses in early puberty leads to the augmentation of nocturnal LH pulses in children, before any physical development of Tanner genital or breast stage 2 can be observed clinically.^41^ This period may thus be seen as the hormonal onset of puberty. The difference between daytime and overnight LH concentrations persists until the late stages of puberty.^42^ Mean LH and FSH concentrations both increase gradually through pubertal development in concert with sex steroid concentrations, although LH rises to a greater extent, due to differences in feedback mechanisms and sensitivity for these two hormones, as described above. There is both an increase in basal concentrations of LH and FSH and a greater number and amplitude of LH peaks.^43^ The negative feedback from gonadal steroids on the hypothalamic–pituitary production of GnRH and gonadotropins develops by mid‐puberty and becomes dominant over the central inhibitory feedback drive.

In males, plasma testosterone concentrations increase dramatically from the onset of puberty to its completion, in parallel with an increase in testes volume, which is not only due to the increasing number of germ cells but also Sertoli cells (Table 2).^44^ In early puberty, testosterone may only be detectable in the early morning but this pronounced diurnal rhythm in testosterone in early and mid‐puberty gradually declines with age, mirroring the day–night ratios of gonadotropins.^45^

**Table 2:** Serum gonadotropins, gonadal and adrenal steroids in stages of pubertal development in (a) girls and (b) boys (with respective testis volumes)

Girls				
Tanner stage	LH (IU/L)	FSH (IU/L)	Estradiol (pmol/L)	DHEAS (µmol/L)
1	0.01–0.21	0.50–2.41	5–10	<0.5
2	0.27–4.21	1.73–4.68	5–115	
3	0.17–4.12	2.53–7.04	5–180	
4	0.72–11.01	1.26–7.37	25–345	
5	2.4–12.6	3.5–12.5	45–854	1.6–7.8

Boys					
Tanner stage	Testicular volume (ml)	LH (IU/L)	FSH (IU/L)	Testosterone (nmol/L)	DHEAS (µmol/L)
1	<4	0.02–0.42	0.22–1.92	<0.5	<0.5
2	4–8	0.26–4.84	0.72–4.60	0.4–2.4	
3	8–10	0.64–3.74	1.24–10.37	2.1–9.5	
4	10–20	0.55–7.15	1.70–10.35	4.9–17.9	
5	20–25	1.7–8.6	1.54–12.4	11.1–26.9	2.3–10

*Note*: Values adapted from Knorr et al.^46^ and Barts Health biochemistry data.

Abbreviations: DHEAS, dehydroepiandrosterone sulphate; FSH, follicle‐stimulating hormone; LH, luteinizing hormone.

Girls enter puberty on average about a year earlier than boys: pubertal onset (defined as the transition from Tanner breast stage 1 to stage 2) in healthy girls occurs between 8 and 13 years of age, while in healthy boys, the spectrum of pubertal onset (with the attainment of the testicular volume of 4 ml or greater, Tanner genital stage 2) is between 9 and 14 years. This consensus on the age limits for the definition of normal pubertal timing has remained amongst European clinicians despite the observed trend towards a decreasing age of breast development in girls in the developed world.^47^

## 3 DISORDERS OF PUBERTY

### 3.1 Precocious puberty

Premature sexual maturation is a frequent cause of concern for parents, and for referral to paediatric clinical services. Precocious puberty is defined as the development of Tanner breast stage 2 in girls before the age of 8 years, and of Tanner genital stage 2 (testes volume > 3 ml) before the age of 9 years in boys.^48^ However, confirmation of this clinical diagnosis with biochemical evidence of a rise in gonadotropin and sex steroid concentrations, so demonstrating activation of the HPG axis, is required. Moreover, while precocious puberty in boys is more rarely idiopathic and thus always requires investigation, in girls, there may be slowly progressive forms of precocious puberty that may not necessitate intervention. This distinction in girls, between mild or benign forms of precocious HPG activation and more rapidly developing cases that require treatment, can be challenging and can require repeated basal and stimulated hormonal profiling, as well as monitoring of pubertal development and growth. As a general rule, the earlier the age that puberty commences, the more likely is it to identify an organic cause.^48^

Precocious puberty is most commonly gonadotropin‐dependent, caused by central activation of hypothalamic GnRH pulsatility. It is around five times more common in girls than boys,^49^ and in girls, most commonly due to an unknown or idiopathic aetiology. Much more rarely, peripheral (gonadotropin‐independent) precocious puberty occurs due to autonomous activation of gonadal hormone production, for example, due to McCune‐Albright syndrome, genetic mutations in the LHCGR causing constitutive activation or ovarian cysts secreting estradiol (Table 3). Central precocious puberty usually has a hormonal pattern similar to that of normal puberty, with a pubertal progression that is symmetrical (i.e., consonant), while in peripheral precocious puberty, there may be asynchronous development of Tanner stages.

**Table 3:** Causes of precocious puberty

Girls		Boys	
Central	Peripheral	Central	Peripheral
Idiopathic	Secondary to adrenal androgens, for example, congenital adrenal hyperplasia	Benign or malignant lesion of CNS, for example, astrocytoma, glioma	Secondary to adrenal androgens, for example, congenital adrenal hyperplasia
Genetic, for example, MKRN3 mutations	McCune‐Albright syndrome	H–P damage, for example, radiotherapy, birth asphyxia, hydrocephalus	Testotoxicosis (activating mutation of the LHCGR)
Hypothalamic hamartoma or other CNS lesion or damage	Ovarian cyst or ovarian/adrenal tumour	Genetic, for example, MKRN3 mutations	McCune‐Albright syndrome
		Idiopathic	Testicular or adrenal tumour

Abbreviations: CNS, central nervous system, H–P, hypothalamic–pituitary; LHCGR, luteinizing hormone/chorionic gonadotropin receptor.

#### 3.1.1 Biochemical diagnosis of precocious puberty

The biochemical diagnosis of central precocious puberty is based on pubertal serum gonadotropin concentrations either as basal levels or after stimulation. Basal gonadotropin concentrations are often informative, particularly basal serum LH (Table 4), although it should be noted that all cut‐offs are dependent on the assay used and clinicians should liaise with their local clinical biochemistry department to determine the appropriate threshold for diagnosis. Before the onset of puberty, FSH is usually the predominant gonadotropin, while during and after puberty LH concentrations are higher than FSH. Basal LH is much more sensitive than basal FSH concentration and so is the key to diagnosis, especially if measured by an ultrasensitive assay, such as an immunochemiluminometric (ICMA) assay.^50^ Prepubertal LH concentrations are less than 0.1 IU/L, so LH assays should have a detection limit close to 0.1 IU/L. In cases of central precocious puberty, basal LH concentration usually is ≥0.3 IU/L. An elevated basal LH has high sensitivity and specificity for the diagnosis of precocious puberty in males if an ICMA assay is used,^51^ but is less sensitive in females.^52^ Thus, in girls, basal LH measurement may be adequate to confirm but not to refute the diagnosis of central precocious puberty.^53^ However, if basal gonadotropin concentration measurements are not clear‐cut then a stimulation test involving a single injection of GnRH can be used.^54^ The response to GnRH stimulation is considered the gold standard for the diagnosis of central precocious puberty, with a pubertal serum LH concentration after stimulation of ≥5 IU/L.^53^ As over recent years the availability of recombinant GnRH has been limited, GnRH analogues have also been considered for the investigation of peak LH and FSH concentrations following stimulation testing.^55^, ^56^ FSH is less informative than LH, but a stimulated LH/FSH ratio of more than 0.66 may help to distinguish progressive from nonprogressive cases not requiring intervention.

**Table 4:** Values of basal LH, estradiol, testosterone, stimulated LH and the stimulated ratio of LH/FSH for diagnosis of puberty

Predictors of puberty	Sensitivity	Specificity	PPV	NPV
Basal LH > 0.3 IU/L^a^ (males)	100%	100%	100%	100%
Basal LH > 0.1 IU/L (females)	67%	100%	100%	63%
Stimulated LH > 5 IU/L^b^	73%	100%	100%	80%
Basal LH/FSH > 1 (males)	100%	100%	100%	100%
Basal LH/FSH > 1 (females)	10%	100%	100%	39%
Stimulated LH/FSH > 1^b^ (males)	100%	100%	100%	100%
Stimulated LH/FSH > 1^b^ (females)	50%	100%	100%	53%
Stimulated LH > 5 IU/L^b^ and basal LH/FSH > 1	61%	100%	100%	56%

*Note*: Table modified from Yazdani et al.^52^ using data from Resende et al.^51^

Abbreviations: FSH, follicle‐stimulating hormone; LH, luteinizing hormone; NPV, negative predictive value; PPV, positive predictive value.

^a^ Measured by immunochemiluminometric assay.

^b^ Concentration 1 h after leuprolide administration.

In terms of sex steroid measurements, in boys, testosterone is assessed with a sensitive method, such as radioimmunoassay (RIA) or liquid chromatography with tandem mass spectrometry, which is a good marker of testicular maturation. In girls, estradiol can be uninformative, firstly because unless there is a very sensitive assay such as RIA it may be undetectable even in cases of true precocious puberty, and secondly because there is overlap between the normal range of estradiol for prepubescent and pubescent girls. Thirdly, the increase in estradiol concentration is also highly variable due to the fluctuation and intermittent secretion of this hormone. Oestrogenic exposure can also be assessed by pelvic ultrasound scans to visualize the mitogenic effect of estradiol on the uterus and ovaries.

However, markedly raised estradiol concentrations may be seen in cases of peripheral precocious puberty such as with McCune‐Albright syndrome or ovarian disease due to cysts or tumours.^57^ In peripheral precocious puberty, the high serum sex steroid concentration is seen in combination with low basal and peak serum LH concentrations after GnRH stimulation, advanced bone age and an estrogenized uterus on ultrasound scan for girls. Measurement of hCG, alpha fetoprotein and other tumour markers is warranted if peripheral precocious puberty is suspected. It is important to remember that excess steroid production from the gonads or adrenal glands may secondarily promote the activation of the HPG axis, with resultant central precious puberty.

Boys with precocious pubertal development and girls with definite central precocious or peripheral precocious puberty need further investigations, including assessment of bone age (which is usually advanced in patients with progressive precocious puberty), pelvic or testicular ultrasound scans and brain magnetic resonance imaging to image the hypothalamus and pituitary glands.

#### 3.1.2 Distinguishing true precocious puberty from benign variants

Variants of normal puberty may be difficult to distinguish from precocious puberty.^58^ In girls aged 2–3 years, the dominance of pulsatile FSH secretion following mini‐puberty can stimulate low levels of oestrogen secretion with breast budding, a condition known as premature thelarche. Premature adrenarche occurs due to androgen production from the adrenals, which stimulates pubic and axillary hair development, apocrine sweat gland activity with the development of body odour, and can be associated with a moderate increase in growth rate or advance in bone age with modest signs of hyperandrogenism for example, mild acne. Premature adrenarche is generally considered a benign condition as endogenous puberty occurs at a normal time without the need for treatment, and adult height is within the expected range for parents. However, associations between premature adrenarche and increased body mass index, central precocious puberty and PCOS have been observed.^59^ Girls with higher circulating concentrations of adrenal androgens, such as dehydroepiandrosterone sulphate and androstenedione, tend to develop B2 at an earlier age, as well as having an earlier age of menarche and lower AMH levels.^60^ Finally, in older girls, at least 50% of cases of premature sexual maturation will regress or stop progressing without the need for treatment.^61^

### 3.2 Delayed, arrested or absent puberty

Delayed puberty is generally defined in girls by a lack of Tanner stage 2 breast development by the age of 13 years, or by the absence of menarche at the age of 15 years, and in boys by the lack of Tanner genital stage 2 (testicular volume above 3 ml) at the age of 14 years.^62^ In an adolescent with delayed puberty the main differential diagnosis is between a central or gonadal cause.

#### 3.2.1 Hypergonadotropic hypogonadism

Primary gonadal disorders display a biochemical picture of hypergonadotropic hypogonadism at puberty. During mid‐childhood, serum gonadotropins may be similar or mildly higher than those in normal controls. In boys, the most common underlying condition is Klinefelter syndrome (47XXY), but in boys with this condition puberty usually starts within a normal timeframe; but tends to arrest in mid‐puberty with rising FSH and falling testosterone concentrations.^63^ In boys, low serum inhibin B reflects primary germ cell failure. Gonadotropin concentrations assessed by basal LH and FSH determination are often increased by 10 years of age in females in primary ovarian insufficiency or in Turner syndrome (45X), but in a prepubertal child hypogonadotropic hypogonadism does not exclude primary gonadal disorders. Women with Turner syndrome without mosaicism commonly do not enter puberty, but overall, 30% of women with a diagnosis of Turner syndrome will undergo some spontaneous pubertal development, and 2%–5% have spontaneous menses and may have the potential to achieve pregnancy without medical intervention.^64^ Other causes of hypergonadotropic hypogonadism in adolescence are described in Table 5.

**Table 5:** Differential diagnoses of hypergonadotropic hypogonadism in adolescence

Females	Males
Turner syndrome (45X)	Klinefelter syndrome (47XXY)
Premature ovarian insufficiency	Congenital anorchia/testicular regression
Disorders of sexual development/gonadal dysgenesis	Mumps orchitis, coxsackie virus
Chemotherapy/radiation therapy	Disorders of sexual development/gonadal dysgenesis
Galactosaemia	Chemotherapy/radiation therapy

#### 3.2.2 Hypogonadotropic hypogonadism

A hormonal picture of hypogonadotropic hypogonadism in adolescence is most commonly due to self‐limited or constitutional delayed puberty,^65^ seen in 83% of boys and 30% of girls with pubertal delay.^66^ In this condition the HPG axis remains dormant beyond the classical age limits for pubertal onset in the healthy population, and young people present with prepubertal or early pubertal Tanner staging, low sex steroid concentrations and undetectable or low gonadotropins. Classically, this is a benign condition where individuals will go through puberty, either after a period of time or after a short course of exogenous sex steroids, and then go on to have a healthy reproductive lifespan. However, it can be difficult to distinguish this diagnosis from the more severe causes of delayed puberty with low gonadotropins, including isolated hypogonadotropic hypogonadism. Furthermore, patients may have such delayed pubertal development that this has significant sequelae for adult height and psychosocial development.^67^ Finally, it is increasingly recognized that there is a spectrum of gonadotropin deficiency and that some patients previously thought to have ‘isolated’ delayed puberty may have underlying HPG axis defects with repercussions for later health and fertility.^68^

Delayed puberty with low gonadotropin concentrations may also be due to functional hypogonadism or to congenital or acquired GnRH deficiency, leading to hypogonadotropic hypogonadism. Functional hypogonadism with delayed puberty, arrested puberty or functional (hypothalamic) amenorrhoea is seen in young people with chronic disease (renal, liver, respiratory, cardiac or inflammatory amongst others), poor nutrition, including anorexia nervosa and excessive exercise.^69^ Therapy for the underlying condition can result in normalisation of the HPG axis.

**Figure 3:**
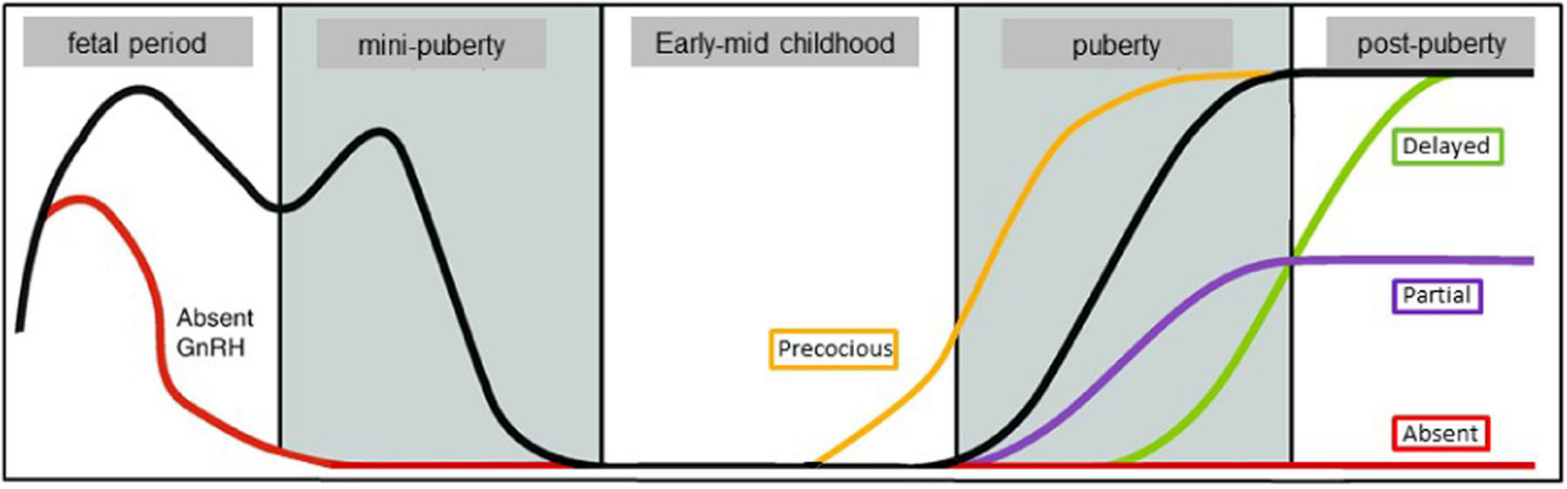
GnRH secretion from prenatal life through to the postpubertal period, in individuals with healthy puberty (black line), precocious puberty (yellow), delayed puberty (green), partial hypogonadotropic hypogonadism (purple) and complete hypogonadotropic hypogonadism (red). GnRH, gonadotropin‐releasing hormone [Color figure can be viewed at wileyonlinelibrary.com]

Congenital hypogonadotropic hypogonadism leads to absent, partial or arrested pubertal development in adolescence but may also be apparent in males in infancy with micropenis and cryptorchidism, due to GnRH deficiency during foetal development (Figure 3). Mini‐puberty provides a window of opportunity for evaluation of the functionality of the HPG axis in males with hypogonadism before puberty.^70^ Hypogonadotropic hypogonadism may be: idiopathic; associated with anosmia when it is termed Kallmann syndrome; associated with an anatomical defect or other hormonal deficiencies of the hypothalamus or pituitary; or acquired due to tumour, radiotherapy, trauma or vascular events.^71^ There are numerous other syndromic conditions associated with hypogonadotropic hypogonadism (Table 6), including *IGSF1* deficiency, which results in a syndrome of X‐linked central hypothyroidism with delayed puberty and macro‐orchidism in male patients, reflecting their relative FSH excess.^72^

**Table 6:** Syndromic associations with CHH or KS

Gene	OMIM ID	CHH	KS	Syndrome/syndromic features
FGFR1/FGF8	136350/600483	x	x	Hartsfield^73^
LEP/LEPR	164160/601007	x		Severe obesity^74^, ^75^
PCSK1	162150	x		Obesity, ACTH deficiency, diabetes^76^
DMXL2	616113	x		Polyendocrinopathy polyneuropathy syndrome^77^
RNF216/OTUD4	609948/611744/212840		x	Gordon Holmes^78^
PNPLA6	603197		x	Gordon Holmes, Oliver Mcfarnlane,^79^ Lawrence Moon^80^
SOX10	602229		x	Wardenburg^81^
CHD7	608892	x	x	CHARGE^82^
POLR3A/POLR3B	614258/614366	x		4H^83^
NR0B1	300473	x		Adrenal hypoplasia^84^
REV3L/PLXND1	157900	x	x	Moebius syndrome^85^
15q11.2	176270	x		Prader Willi^86^

Abbreviations: ACTH, adrenocorticotrophic hormone; CHH, congenital hypogonadotropic hypogonadism; KS, Kallmann syndrome.

#### 3.2.3 Hypothalamic dysfunction

A number of hypothalamic disorders, additional to the ones discussed, can lead to disturbance of gonadotropin secretion during or after puberty. PCOS is classically associated with raised serum LH concentrations, elevated ovarian androgens and hyperinsulinemia. A rapid frequency of GnRH pulses leads to increased LH pulse frequency and amplitude and a relative FSH deficiency, with oligo‐ or anovulation.^28^ Diagnosis in adolescence is challenging as irregular menstrual cycles are common in the year following menarche and AMH concentrations and pelvic ultrasound morphology are not reliable diagnostic features in this age group.^87^ Girls with PCOS can also present with delayed menarche. Primary amenorrhoea in PCOS is rare, and LH concentrations are similar between girls with PCOS and either primary or secondary amenorrhoea.^88^

In contrast, a relative LH deficiency, as seen in partial hypogonadotropic hypogonadism, is found in hypothalamic amenorrhoea and hyperprolactinaemia. Both of these conditions are associated with oligo‐ or amenorrhoea, anovulation and a reduction in LH pulse frequency.^28^ Congenital hypothalamic disorders or acquired dysfunction, for example due to tumours can also affect GnRH pulsatility, with slow pulses leading to relative LH deficiency and favouring FSH production. Thus, hypothalamic conditions can result in either precocious or delayed puberty onset or disrupted menstrual cyclicity.

#### 3.2.4 Biochemical diagnosis of hypogonadism

**Figure 4:**
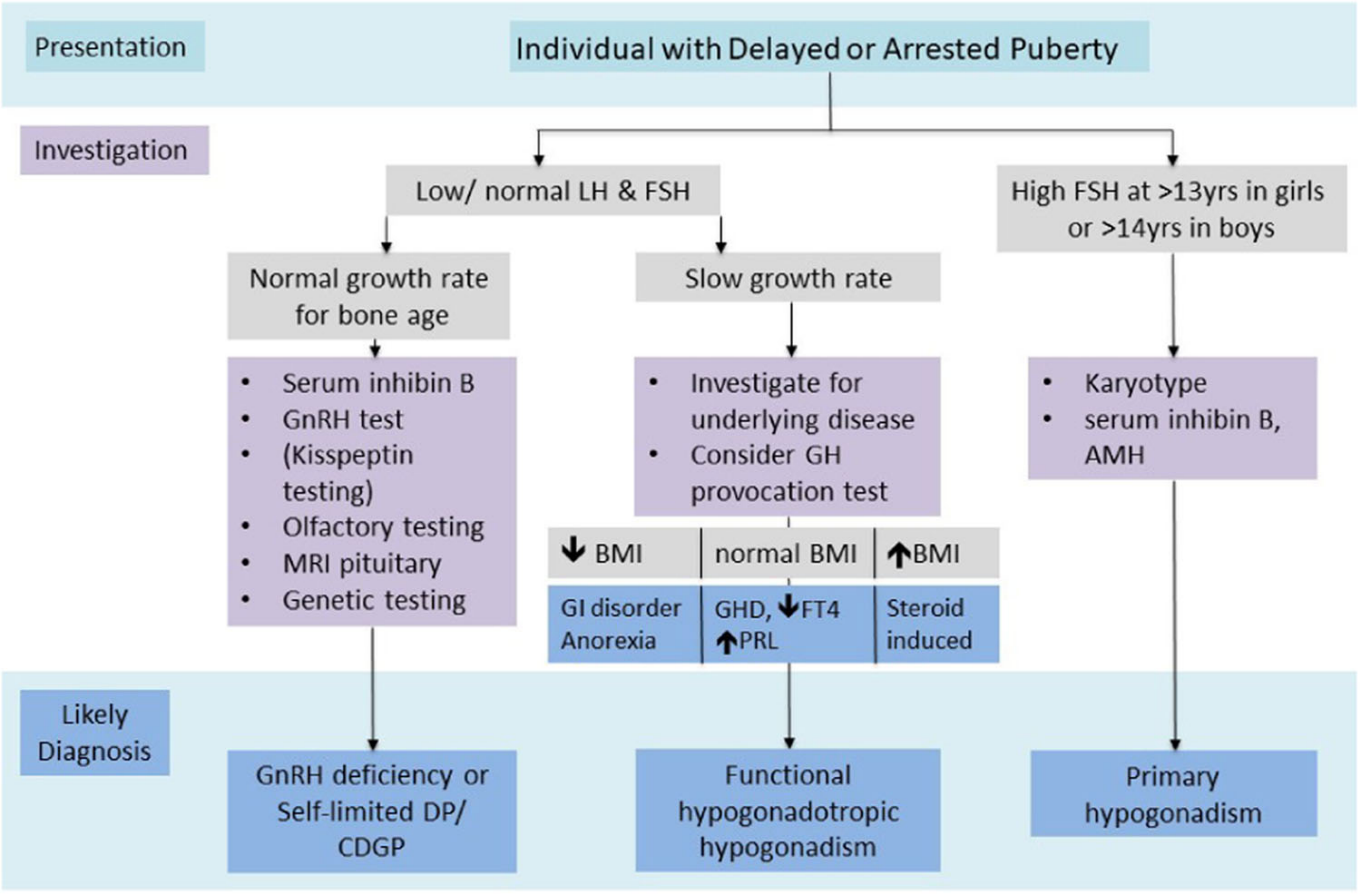
Investigation flow chart for individuals presenting with delayed puberty (DP) in adolescence. BMI, body mass index; FT4, free thyroxine; GH, growth hormone; GHD, growth hormone deficiency; GI, gastrointestinal; PRL, prolactin [Color figure can be viewed at wileyonlinelibrary.com]

After initial clinical assessment, biochemical investigations in an adolescent with delayed puberty (Figure 4) should include basal LH and FSH (to look for hypergonadotropic hypogonadism) with sex steroid measurements, screening for asymptomatic systemic illness (full blood count, erythrocyte sedimentation rate, renal function, coeliac screen, liver function, electrolytes), thyroid function tests, insulin‐like growth factor 1 and prolactin. A karyotype is important in females with primary hypogonadism. Investigation of the differential diagnosis between self‐limited delayed puberty and hypogonadotropic hypogonadism in adolescents who present with delayed puberty is often more difficult because both conditions may present with the same clinical and hormonal features. A number of physiological and stimulation tests are used to try to distinguish these phenotypes, including gonadotropin response to GnRH, testosterone response to hCG and first morning‐voided urine testosterone, FSH and LH.^89^, ^90^ In research settings, assessment of LH pulsatility by frequent sampling and prolactin response to provocation have been used.^91^

Both basal gonadotropin concentrations and GnRH stimulation tests have limited specificity for the following reasons: Basal gonadotropins are generally low in prepubertal patients whether their sexual immaturity is due to age, constitutional delay or underlying GnRH deficiency; a prepubertal response to a GnRH stimulation test may be seen in a patient with self‐limited delayed puberty who has the capacity to progress well through puberty in response to short‐course sex steroid therapy, and hence does not predict future reproductive capacity; and a pubertal response to GnRH stimulation test may be falsely reassuring in a patient with hypothalamic GnRH deficiency, who has intact pituitary function and can respond to exogenous GnRH but will not enter puberty spontaneously. Several studies have proposed diagnostic flowcharts to aid with the investigation of patients with hypogonadotropic delayed puberty.^91^, ^92^ Despite this, the clinical follow‐up to assess spontaneous pubertal development or response to sex steroid therapy is often warranted before a definitive diagnosis can be made.^93^

Stimulation tests using more potent GnRH agonists or hCG (in males) may be useful to discriminate these conditions but are not always clinically practical to perform.^94^ Basal inhibin B is the most promising biochemical investigation in terms of its sensitivity, with studies reporting a threshold of less than 35 pg/ml in prepubertal boys to discriminate permanent hypogonadotropic hypogonadism from self‐limited delayed puberty,^95^ but this utility has not been demonstrated in girls. In males, the trio of testes volume (cut‐off: 1.1 ml), maximal stimulated LH (cut‐off: 4.3 IU/L) and basal inhibin B concentration (cut‐off: 61 pg/ml) have been proposed as the most effective discriminator between these two conditions.^65^ Most recently, FSH‐stimulated inhibin B, at a cut‐off of 116.14 pg/ml in males and 116.50 pg/ml in females was shown to have a 100% sensitivity and specificity for prediction of entry into puberty.^96^ The diagnostic utility of basal and stimulated inhibin B needs further confirmation in clinical studies.

To assess GnRH production by the hypothalamus, LH measurement in response to stimulation with kisspeptin has been proposed as a useful test to identify individuals with GnRH deficiency and thus permanent hypogonadotropic hypogonadism. Kisspeptin stimulates GnRH pulsatility, and thus promotes LH, and to a lesser extent FSH, secretion. Inactivating mutations of *KISS* and *KISS1R* genes have demonstrated the importance of the KISS regulatory system in the regulation of human puberty and fertility, while activating *KISS1R* mutations have resulted in precocious puberty.^97^ A recent clinical study found that maximal LH rise after kisspeptin administration was more accurate for diagnosis of men with GnRH deficiency than GnRH stimulation testing.^98^ In a parallel study in adolescents with pubertal delay (3 females and 13 males), peak LH post kisspeptin stimulation was demonstrated to be superior to GnRH stimulation testing for predicting capacity to progress through puberty.^99^ All eight study participants with a maximum LH response to kisspeptin of ≤0.4 IU/L reached age 18 years without developing physical signs of puberty, thus confirming the diagnosis of hypogonadotropic hypogonadism. While further research is required to delineate the parameters of using kisspeptin in clinical paediatric practice, this is a promising area for the biochemical diagnosis of GnRH deficiency in adolescence.

## 4 CONCLUSION

Using the interpretation of reproductive hormones in late childhood and adolescence to distinguish healthy puberty from its disorders is not straightforward. The biochemical parameters must be taken in the context of clinical features, imaging and radiological studies, and monitoring of pubertal progression. Diagnosis may require more extended or resource‐intensive investigations, such as measurement of gonadotropin response to stimulation with GnRH or, more recently, kisspeptin, as well as genetic analysis with whole‐exome or panel testing.^100^ The expertize of the clinical team to put together these pieces of the jigsaw puzzle is key, to allow appropriately directed management in a limited‐time window, to minimize negative outcomes and optimize therapeutic care for our patients.

## CONFLICT OF INTERESTS

The author declare that there are no conflicts of interest.
